# Assessment of Physical, Technical, and Tactical Analysis in the Australian Football League: A Systematic Review

**DOI:** 10.1186/s40798-022-00518-8

**Published:** 2022-10-08

**Authors:** Andrew Vella, Anthea C. Clarke, Thomas Kempton, Samuel Ryan, Aaron J. Coutts

**Affiliations:** 1 Sport and Exercise Science, La Trobe University, Bundoora, VIC Australia; 2 Carlton Football Club, Melbourne, VIC Australia; 3 School of Sport, Exercise and Rehabilitation, Faculty of Health, Human Performance Research Centre, University of Technology Sydney (UTS), PO Box 123, Broadway, NSW 2007 Australia

**Keywords:** Match activity, Player movement analysis, Contextual factors, Technical match events, Tactics, Integrated data

## Abstract

**Background:**

Elite Australian Football (AF) match-play requires proficiency in physical, technical, and tactical elements. However, when analysing player movement practitioners commonly exclude technical and tactical considerations, failing to recognise the multifactorial nature of AF match-play and providing little context into the movement requirements of the players.

**Objectives:**

This systematic review aimed to identify the physical, technical, and tactical requirements of the Australian Football League (AFL) and to highlight the importance of integrating data from multiple sources when analysing player output.

**Methods:**

A systematic search of electronic databases (CINAHL, PubMed, Scopus, SPORTDiscus, and Web of Science) was conducted from January 2009 to June 2022. Keywords relating to physical, technical, and tactical match requirements were used.

**Results:**

Forty-eight studies met the inclusion criteria. In isolation, physical requirements were the most analysed construct within the AFL (*n* = 17), followed by technical (*n* = 9) and then tactical (*n* = 6). Thirteen studies integrated physical and technical elements, one study integrated technical and tactical elements, one study integrated physical and tactical elements, and one study integrated all three elements. Movement analysis centred around average ‘whole’ match requirements, whereas technical and tactical match analyses focused on key performance indicators of match performance.

**Conclusion:**

While the physical requirements of the AFL have been well documented, there is little understanding of how player technical output and various team tactics influence player movement requirements. Knowledge of how the elements of AF match-play interact with one another could enhance our understanding of match performance and provide a greater resource for training prescription.

## Key Points

- Match running requirements are reliant on numerous technical and tactical contextual factors that are uniquely dependent on the team, opposition, and the AFL season/s in which the study was conducted (e.g. rule changes).
- While there is an abundance of information on the global and peak running requirements of elite male AF players, there is limited information on how both technical and tactical factors influence match running requirements.
- The quantification of how the technical, tactical, and physical elements of match-play are related is important for understanding the requirements of athletes during competitive matches, for player analysis, and to inform training drill design.

## Introduction

Australian Football (AF) is a contact field-based sport characterised by intermittent locomotive activity, where bouts of high-intensity activity (running, accelerating, and sprinting) are interspersed with prolonged low-intensity activity (walking and jogging) [1, 2]. The game is contested between two teams of 18 players, with four players available for interchange, with a maximum of 90 rotations permitted per team throughout the match [2]. Competition matches are divided into four 20-min quarters (plus added time for stoppages), separated by two 6-min quarter breaks and a 20-min half time break [3]. The objective of the game is to outscore the opposing team, which is achieved by moving the ball to a scoring position through the collective effort of the team [1–3]. The premier men’s competition is the Australian Football League (AFL) where 18 teams play 22 home-and-away matches followed by a four-week finals series for the eight top-ranked teams to determine the premiership [3]. There are three key performance elements in AF. They are: physical (e.g. running, accelerating, walking) [4, 5], technical (e.g. kicking, handballing, tackling) [6, 7], and tactical (e.g. collective team behaviour, ball movement) [8, 9]. Although these have often been studied separately, they are symbiotic to one another. On this basis, coaches and scientists are encouraged to undertake multifactorial analysis of match-play to encompass the combination of physical, technical, and tactical elements.

Technological developments have led to global positioning systems (GPS) devices becoming prominent in sports, allowing practitioners to quantify the activity completed by athletes. The first published study using GPS technology in sport was prior to the turn of the century [10], and in 2005 the AFL widely adopted the microtechnology for player monitoring during training and match play [1, 11]. In contemporary AFL, it is mandatory that all players wear GPS units during all formal matches and training sessions. This provides real-time and post hoc information on the external load completed by players, which is used in training and match analysis and to inform future training prescription [12]. GPS is typically classified by their sample rate (expressed in Hertz) at which the chipset and satellite communicate per second to determine the device’s location. The first devices used in the AFL sampled at 1 Hz (one sample per second); however, with advancements in technology commercially available GPS units now come with sample rates of 5 Hz, 10 Hz or 15 Hz [3, 13]. These devices have enabled the quantification of player activity demand (i.e. distance, running velocities, accelerations, and peak movement demands) during match-play, with GPS units with greater sampling rates providing more accurate data [13, 14]. While most research observing physical match activity is with the intention to inform training prescription [5, 15], other research is starting to explore the link between physical movements and match outcomes at a team level [16, 17], and the link between physical movements and technical involvements during match-play [18].

Skill execution is an important contributor to individual and team performance in AF [17, 19] and is typically quantified by the number and efficiency of key technical actions (e.g. kicks, handballs, and marks). In the AFL, these match events are collected by a commercial statistics provider (Champion Data Pty Ltd, Melbourne, VIC), assisting in match analysis and training drill design [20–22]. While an abundance of information exists regarding technical requirements during AFL match-play, there is limited information regarding skill-based match events that influence match activity requirements [17, 23]. Research that has combined both physical and technical measures of AFL match-play typically centres around how both elements influence individual and team performances [17, 23]. These studies provide an understanding of how technical skill-based measures have a greater impact on performance when assessed via subjective (e.g. coach’s player rating) and objective (e.g. Champion Data player rank) measures [17, 23]. However, in regard to how these two elements interact with one another, evidence is limited. To provide more insightful information on player activity profiles, technical and tactical data may be integrated, providing match-play context such as the field location and phase of play (e.g. attack, defence, in-dispute) in which players were directly involved in the play. By understanding the relationship between technical skill involvements and activity requirements in AF, coaches can design training drills that are representative of match-play, serving to not only refine the players’ technical proficiency but to additionally refine the skills necessary to both practice and implement specific tactical plays/styles.

Understanding the tactical behaviours and interactions (i.e. player positioning and passing networks) of a team is crucial to understanding individual match activity requirements given the complexity of team sport performance. Without the context of the team, the technical output of individual players in isolation is not enough to gain meaningful information about a player’s physical output as it is the tactics employed (e.g. fast/slow ball movement) during various stages of match-play that provide context to the activity profiles of the athletes being assessed [2, 24, 25]. Complex and social network analysis in sports examines the interaction between players, and how certain characteristics of teamwork relate to performance [2, 26]. Spatiotemporal data on the other hand examine team behaviour and its relationship to performance by analysing the collective positioning of players during match-play [27–30]. Spatiotemporal data derived from GPS units and network analysis methods have therefore been employed to observe the interactions and positioning of players throughout a match, providing insights into the tactics of various teams [24, 26–32]. While most research using these techniques has been conducted in soccer [24, 27–29], there has been a recent shift towards these analytical approaches in AF [30–32]. With the similarity between AF and soccer—both being 360° games where players can pass in any direction—similar analysis methods may be useful in understanding the collective actions and interactions amongst teammates during AF match-play. Knowledge of the influence team tactics have on activity profiles may inform training design and team tactics during match-play. This understanding could assist coaches by providing insights into the physical capabilities required to execute the desired game plan under the stressors of AFL competition.

While the activity requirements of AFL match-play have been well documented, these analyses are often isolated from technical and tactical considerations, two important elements of match-play [33]. Given the multifactorial requirements of AF, it is important to incorporate all three elements (physical, technical, and tactical) when trying to understand the activity requirements of athletes during competitive matches. Therefore, further investigation of how all three elements interact during match-play is warranted. The aims of this systematic review were to (1) provide an update of match activity requirements as most commonly examined in the AFL (i.e. absolute and relative distances for average, high speed, and peak activity requirements); (2) detail the technical requirements of AFL match-play; (3) identify common tactical analysis methods of match-play, and (4) identify research that has integrated the three elements (physical, technical, and tactical) in AFL and how these influence one another.

## Methods

### Design and Search Strategy

This systematic review was conducted in accordance with the PRISMA (Preferred Reporting Items for Systematic Review and Meta-analysis) guidelines [34]. A systematic search of the literature was conducted in various electronic databases: CINAHL, PubMed, Scopus, SPORTDiscus, and Web of Science. Articles for this review were focused on peer-reviewed journals from January 2009 until June 2022. The start date was chosen based on when GPS became prominent in the AFL and all teams were regularly using GPS to monitor player workloads [11]. The combination of the terms listed in Table 1 was used to search and obtain the titles, abstracts, and key words of articles within each database.

**Table 1:** Search terms used in each database. Searches 1 and 2 were combined with “AND”

Search 1	Search 2
“Australian Football” OR “Australian Football League” OR “Australian Rules Football”	“Match demands” OR “activity profiles” OR “running demands” OR “game demands” OR “running performance” OR "external load" OR "contextual factors" OR "movement patterns" OR "team behaviour" OR "skill" OR "technical" OR "match outcome" OR “skill measures” OR “tactics” OR “performance indicator” OR “match performance” OR “network analysis” OR “match event” OR “spatiotemporal”

### Screening and Study Selection

All references obtained were imported into a reference manager application (Endnote X9, Thomas Reuters, Philadelphia, USA) where all duplicate articles were then eliminated. Articles were screened independently by two researchers (AV and TK) to decide which studies met the inclusion criteria determined by the title, abstract, or when required via full text. The titles and authors were not masked to the reviewers.

Studies that assessed the physical (via GPS), technical, or tactical elements of AFL competition were included in the review. The exclusion criteria of this review included any book, video conference, or review article. Furthermore, any study that assessed musculoskeletal injuries or the psychological, sociological, or nutritional aspect of AFL was additionally excluded. Likewise, any study examining the physical, technical, or tactical demands of training, or any competition other than the AFL (e.g. youth, state league, or women’s AF) was excluded. To avoid artificially high match running intensities, articles that reported data for athletes that played < 70% game time were excluded from the review [16]. Upon selecting the articles for inclusion, the reference list of each article was scanned for any potentially relevant studies that were not retrieved in the original search.

### Data Extraction

For all studies included in this systematic review, data characteristics (i.e. number of files/matches/players) and methods of data collection and analysis were extracted by one researcher (AV). Where studies included the use of GPS, data on GPS unit specifications (i.e. brand, model, sampling frequency, software) were also extracted. For the purpose of this review, reporting of GPS data was limited to total distance (TD) (m), relative distance (m·min^−1^), high-speed running (HSR) distance (m), HSR relative distance (m·min^−1^), peak relative distance (m·min^−1^), and peak HSR relative distance (m·min^−1^). All variables were converted to metres and metres per minute for ease of comparison, while HSR thresholds were converted to km·h^−1^. Where data were reported using a different unit of measure, conversion and/or calculation based on total match or active (on-field) playing time duration was completed where appropriate. For example, to calculate relative distance, TD was divided by total match duration in minutes. Additionally, mean and standard deviations (SD) that were presented in figures were extracted using an online extraction tool WebPlotDigitizer v4.2. Where studies examined the technical or tactical aspects of AFL, the data retrieval (e.g. Champion Data, broadcast vision) and analysis method (e.g. social network analysis, spatiotemporal data) were also extracted.

### Assessment of Methodological Quality

The methodological quality of each study was assessed by two researchers using a modified version of a previously validated scale [35]. Certain criteria measures were not applicable to the studies in this review. Therefore, only 11 of the 27 criteria were used (1–3, 6, 7, 10–12, 16, 18, 20). This is a similar approach to other reviews within this field [36]. Question 10 was modified to assess the inclusion of effect size reporting as opposed to probability values (i.e. *p*-values). Using the 11 criteria used in the assessment a score of ‘0’ represented if the item was absent or insufficiently detailed, while a score of ‘1’ represented if the item was explicitly detailed. Methodological quality scores ranged from excellent (10–11); good (8–9); fair (5–7); and poor (< 5). No studies were omitted based on the methodological quality assessment criteria.

### Statistical Analysis

A meta-analysis was not performed as the wide variety of study designs and outcome variables meant studies could not be pooled. All data are presented as mean ± SD or as mean (confidence limits, CL) unless otherwise stated.

## Results

### Search Results

**Fig. 1:**
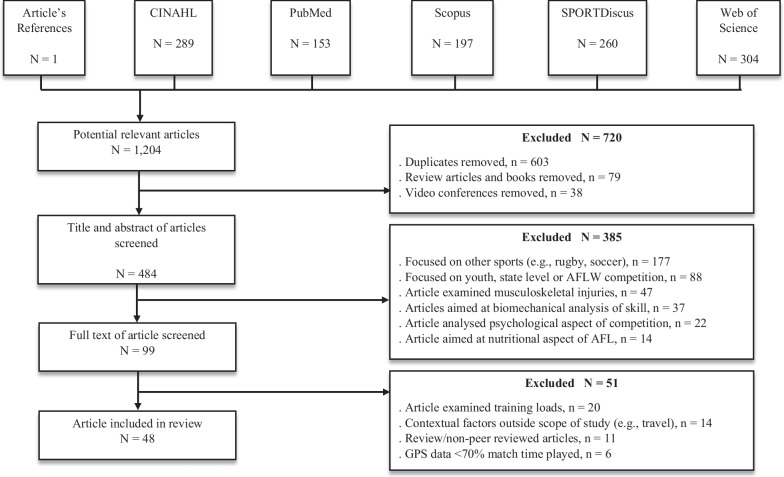
Study selection flow chart

The initial search returned 1,204 articles from across five databases (CINAHL = 289, PubMed = 153, Scopus = 197, SPORTDiscus = 260, Web of Science = 304), with one study added after being identified in the reference list of another article. Following the initial search, 720 articles were removed for being either a duplicate, book, video conference, or review article. The title and abstract of the remaining 484 articles were then screened where a following 385 were removed for not fitting the inclusion criteria. This resulted in 99 articles being screened via full text where a further 51 articles were excluded. In total, 48 articles met the inclusion criteria and were included in this review. The schematic process of articles that were potentially relevant for inclusion is displayed in Fig. 1.

### Methodological Quality

The methodological quality assessment scores of each study are shown in Table 2. Scores ranged from seven to nine for the 11 items assessed. Of the 48 studies, 46% (*n* = 22) received a score of nine, 29% (*n* = 14) received a score of eight, and 25% (*n* = 12) received a score of seven. Studies that received a greater score are more likely to have prevented systematic errors (bias) and provide readers with more critical information to avoid erroneous conclusions [37].

**Table 2:** Characteristics of the studies in this review

Study	Construct analysed	Analysis method	No. of matches	No. of files	No. of players	Methodological quality score
Alexander et al. (2019) [30]	Tact	Spatiotemporal data	1	NR	22	8
Anderson et al. (2018) [55]	Tech	BV	198	NR	NR	7
Aughey (2010) [47]	Phys	GPS	29	147	18	9
Aughey (2011) [49]	Phys	GPS	6	NR	8	9
Aughey (2013) [64]	Phys	GPS	29	2015	35	9
Bauer et al. (2015) [4]	Phys & Tech	GPS & CD	11	204	35	9
Black et al. (2016) [51]	Phys & Tech	GPS & BV	13	163	24	9
Braham & Small (2018) [26]	Tact	CNA	207	NR	NR	7
Brewer et al. (2010) [15]	Phys	GPS	NR	315	33	9
Corbett et al. (2018) [20]	Phys	GPS & LPS	21	NR	39	8
Corbett et al. (2019) [53]	Phys & Tech	GPS, LPS & CD	19	NR	37	8
Coutts et al. (2010) [48]	Phys	GPS	25	79	16	8
Coutts et al. (2015) [5]	Phys	GPS	19	342	39	9
Delaney et al. (2017) [52]	Phys	GPS	30	623	40	9
Dillon et al. (2018) [23]	Phys & Tech	GPS & CD	15	NR	33	9
Esmaeili et al. (2020) [44]	Phys	GPS	207	NR	657	9
Gronow et al. (2014) [50]	Phys	GPS	14	NR	36	8
Hiscock et al. (2012) [41]	Phys & Tech	GPS & CD	17	355	30	9
Ireland et al. (2019) [6]	Tech	CD	16	NR	33	9
Johnston et al. (2012) [38]	Phys & Tech	GPS & CD	12	69	21	9
Johnston et al. (2015) [39]	Phys & Tech	GPS & CD	NR	230	21	9
Johnston et al. (2016) [40]	Phys & Tech	GPS & CD	NR	336	19	9
Johnston et al. (2019) [18]	Phys & Tech	GPS & CD	22	450	38	9
Kelly et al. (2019) [54]	Phys	GPS & CD	NR	237	20	9
Kempton et al. (2015) [56]	Phys	GPS & CD	31	511	33	8
Montgomery & Wisbey (2016) [46]	Phys	GPS & CD	NR	7730	21	9
Mooney et al. (2011) [16]	Phys & Tech	GPS	5	NR	46	8
Mooney et al. (2013) [45]	Phys	GPS	22	NR	15	8
Parrington et al. (2013) [21]	Tech	BV	14	NR	NR	7
Rennie et al. (2020) [61]	Phys & Tech	GPS & CD	18	360	33	9
Robertson et al. (2016) [22]	Tech	CD	39	NR	NR	7
Robertson et al. (2016) [19]	Tech	CD	198	NR	NR	7
Ryan et al. (2017) [42]	Phys	GPS	15	NR	34	9
Sargent & Bedford (2013) [2]	Tact	SNA	25	NR	34	7
Sheehan et al. (2020) [31]	Tact	CNA	73	1603	48	7
Sullivan et al. (2014) [7]	Phys & Tech	GPS & CD	15	292	40	8
Sullivan et al. (2014) [17]	Phys & Tech	GPS & CD	15	292	40	8
Taylor et al. (2020) [60]	Tact	CD & CNA	194	1720	665	8
Varley et al. (2014) [14]	Phys	GPS	27	176	28	8
Vella et al. (2020) [25]	Phys & Tact	GPS	13	NR	35	9
Vella et al. (2022) [62]	Phys, Tech & Tact	GPS & CD	13	NR	35	9
Wisbey et al. (2010) [43]	Phys	GPS	NR	793	179	8
Woods. (2016) [57]	Tech	CD	394	NR	NR	7
Woods et al. (2017) [8]	Tech	CD	249	NR	NR	8
Young et al. (2019) [32]	Tact	SNA	1516	3032	NR	7
Young et al. (2019) [58]	Tech	CD	3145	NR	NR	7
Young et al. (2019) [59]	Tech	CD	3145	NR	NR	7
Young et al. (2020) [9]	Tech & Tact	CD & SNA	1516	3032	NR	7

Phys, Physical; Tech, Technical; Tact, Tactical; GPS, Global Positioning System; LPS, Local Positioning System; BV, Broadcast vision; CD, Champion Data; SNA, social network analysis; CAN, complex network analysis; NR, not reported

### Study Characteristics

Most studies (*n* = 32) evaluated a single construct of AFL match performance (Table 2). In isolation, physical match requirements were reported in 17 studies, technical requirements in nine studies, and tactical requirements in six studies. Thirteen studies reported both physical and technical variables, one study observed technical and tactical elements together, one study analysed the physical and tactical elements in combination, and one study analysed all three elements in combination. The data source and number of files used for each study are reported in Table 2. The majority of studies reporting on the locomotive requirements of players were from a single team, while studies focusing on technical and tactical elements were more likely to include larger datasets. Catapult devices (10 Hz) were the most common equipment used to collect GPS locomotive data, while skill-based match events were most commonly obtained from one commercial statistics provider (Champion Data Pty Ltd, Melbourne, VIC). Three different analysis methods were used to investigate tactical requirements, including: social network analysis (*n* = 3 studies), complex networks (*n* = 3 studies), and spatiotemporal data (*n* = 1 study) (Table 2). Within locomotor AFL studies, most studies (*n* = 25) reported the average whole match running demands, four studies included peak running requirements, and three reported on possession chain running requirements (passages of play that are controlled by a singular team). Six different HSR thresholds were utilised, while four studies did not report the specific HSR threshold used (Table 3).

**Table 3:** High-speed running thresholds and GPS hardware/software specifics utilised by studies in this review

Study	Locomotive requirements	Brand	Model	GPS sampling frequency (Hz)	Software	HSR threshold
Aughey (2010) [47]	Global	Catapult	NR	5	Logan Plus v 4.1	> 15 km/h
Aughey (2011) [49]	Global	Catapult	NR	5	Logan Plus v 4.1	> 15 km/h
Aughey (2013) [64]	Global	Catapult	NR	5	Logan Plus v 4.2.3	> 15 km/h
Bauer et al. (2015) [4]	Global	Catapult	MinimaxX S4	10	Sprint v 5.0.9.2	> 19.8 km/h
Black et al. (2016) [51]	Peak	Catapult	MinimaxX S4	10	NR	> 15 km/h
Brewer et al. (2010) [15]	Global	GPSports	SPI 10	5	GPSports TAS v 1.6.2	> 15 km/h
Corbett et al. (2018) [20]	Global	Catapult	T5 (LPS) and S5	10	Openfield v 1.11.2 – 1.13.1	> 14.4 km/h
Corbett et al. (2019) [53]	Peak	Catapult	T5 (LPS) and S5	10	Openfield v 1.11.2 – 1.13.1	NR
Coutts et al. (2010) [48]	Global	GPSports	SPI 10	1	GPSports TAS v 1.6	> 14.4 km/h
Coutts et al. (2015) [5]	Global	Catapult	NR	10	Sprint v 5.0.6	> 14.4 km/h
Delaney et al. (2017) [52]	Peak	Catapult	MinimaxX S5	10	Openfield v 1.12.0	> 19.8 km/h
Dillon et al. (2018) [23]	Global	Catapult	Optimeye S5	10	Openfield v 1.11.1	> 20 km/h
Esmaeili et al. (2020) [44]	Global	Catapult	Optimeye S5 & T6 (LPS)	10	Openfield v 1.17 & 1.18	> 18 km/h
Gronow et al. (2014) [50]	Global	GPSports	SPI Pro X	5	Team AMS-release	> 14 km/h
Hiscock et al. (2012) [41]	Global	GPSports	SPI Pro X	15	Team AMS-release	> 14 km/h
Johnston et al. (2012) [38]	Global	Catapult	NR	5	NR	> 14 km/h
Johnston et al. (2015) [39]	Global	Catapult	MinimaxX S3 & S4	5 and 10	Sprint v 5.0.9	> 14.4 km/h
Johnston et al. (2016) [40]	Global	Catapult	MinimaxX S3 & S4	5 and 10	Sprint v 5.0.9	> 14.4 km/h
Johnston et al. (2019) [18]	Peak	Catapult	Optimeye S5	10	Openfield v 1.15.0	NR
Kelly et al. (2019) [54]	Global	Catapult	MinimaxX S4	10	Sprint v 5.1.6	> 14 km/h
Kempton et al. (2015) [56]	Global	Catapult	NR	10	Sprint v 5.0.6	> 14.4 km/h
Montgomery and Wisbey (2016) [46]	Global	Catapult	NR	10	NR	NR
Mooney et al. (2011) [16]	Global	Catapult	NR	5	Logan Plus v 4.4.0	> 15 km/h
Mooney et al. (2013) [45]	Global	Catapult	NR	5	Logan Plus v 4.4.0	> 15 km/h
Rennie et al. (2020) [61]	PC	Catapult	Optimeye S5	10	Sprint v 5.1.7	> 14.4 km/h
Ryan et al. (2017) [42]	Global	Catapult	Optimeye S5	10	Openfield v 1.12.2	> 20 km/h
Sullivan et al. (2014) [7]	Global	Catapult	NR	10	Sprint v 5.0.6	> 14.4 km/h
Sullivan et al. (2014) [17]	Global	Catapult	NR	10	Sprint v 5.0.6	> 14.4 km/h
Varley et al. (2014) [14]	Global	Catapult	NR	5	NR	> 19.8 km/h
Vella et al. (2020) [25]	PC	Catapult	Optimeye S5	10	Openfield v 1.22.2	> 20 km/h
Vella et al. (2022) [62]	PC	Catapult	Optimeye S5	10	Openfield v 1.22.2	> 20 km/h
Wisbey et al. (2010) [43]	Global	GPSports	SPI 10 and SPI Elite	1	NR	NR

Global, average requirement; Peak, most intense passages of play; PC, possession chain; LPS, Local Positioning System; NR, not reported

### Match Physical Activity Requirements

#### Total Distance

Studies observing the match distances covered by players typically compared high calibre and low calibre players (based on coaches’ ratings of individual performances), playing positions, and rotation numbers and duration. Players in the AFL cover TD ranging from 11,600 to 13,700 m during a match with a relative distance of 129 ± 10 m·min^−1^ (Table 4). The majority of the match (> 70%) is performed at speeds under the HSR thresholds (Table 4). Three studies examined the differences in physical output between high calibre and low calibre players [38–40]. These studies reported that high calibre players cover greater TD, but similar relative distances to low calibre players [38–40]. Differences in playing positions were examined by seven studies [5, 15, 23, 41–44]. Nomadic players (i.e. midfielders, small forwards, and backs) were reported to cover greater absolute and relative distances [5, 15, 41–44] and were additionally rotated more frequently than key position players (rucks, tall forwards, and backs) [23, 43]. Studies examining the influence of interchange rotations on activity requirements during a match demonstrated that there is an association between the number of rotations a player has and the relative distances covered [23, 42, 44, 45]. Additional studies demonstrated that athletes are better able to sustain relative distance outputs during shorter on-field stints (~ 5 min) compared to longer stints (~ 11 min) [23, 46]. There is conflicting information whether the TD covered by players during a match remains consistent [47] or decreases [44, 48] with each subsequent quarter played. Lastly, two studies reported relative distances are lowest during the early phase of the season [42, 49], with one study reporting an 11% increase in relative distances covered during finals matches [49]. However, this was contrasted in one study which reported a decrease of 1.7% in relative distances covered during finals [44].

**Table 4:** Match running requirements of Australian Football expressed as mean ± standard deviation and mean (95% confidence intervals)

Study	Total distance (m)	Relative distance (m.min^−1^)	HSR distance (m)	HSR relative distance (m.min^−1^)
Aughey (2010) [47]	12,734 ± 1596	127 ± 17	3334 ± 756	34 ± 9
Aughey (2011) [49]	NR	128 (119–138)	3185*	37 (32–42)
Aughey (2013) [64]	NR	140 ± 15	NR	36 ± 14
Brewer et al. (2010) [15]	12,311 ± 1729	128 ± 12	NR	NR
Corbett et al. (2018) [20]	11,608 ± 3573	132*	3198 ± 1,165	36*
Coutts et al. (2010) [48]	12,939 ± 1145	109*	3880 ± 633	33*
Coutts et al. (2015) [5]	12,027 (11,158–12,819)	115 (108–128)	3268 (2598–4314)	32 (25– 43)
Hiscock et al. (2012) [41]	NR	133 ± 12	NR	39 ± 11
Johnston et al. (2012) [38]	13,455 ± 1764	135 ± 12	3045 m*	30 ± 7
Johnston et al. (2015) [39]	13,556 (13,427–13,685)	130 (116–144)	3003*	29 (28–29)
Johnston et al. (2016) [40]	13,556 (13,427–13,685)	130 (116–144)	3003*	29 (28–29)
Kelly et al. (2019) [54]	13,193 (13,047–13,340)	131 (129–132)	3081*	30 (30–31)
Kempton et al. (2015) [56]	13,447 (12,800–14,094)	124 (121–127)	3550 (3300–3800)	33*
Mooney et al. (2011) [16]	NR	139 ± 11	NR	41 ± 10
Mooney et al. (2013) [45]	NR	135 (129–141)	NR	39 (35–43)
Rennie et al. (2020) [61]	12,135 (11,884–12,384)	133 (131–135)	3964 (3830–4097)	33*
Varley et al. (2014) [14]	12,620 ± 1872	129 ± 17	1322 ± 374	14 ± 4
Wisbey et al. (2010) [43]	11,970 + 1900	117*	NR	NR
Mean	12,735	129	3153	33
SD	1212	10	569	6

Data are expressed as means and standard deviations (±); when standard deviation is not presented in study, data are expressed as mean (95% confidence limits)

*NR* not reported

*Denotes when measurements were manually calculated

#### High-Speed Running

Studies reporting on HSR distances covered during AFL match-play typically compare high calibre and low calibre players, playing positions, and the relationship between HSR and match performance. The HSR distances AFL players typically cover throughout a match range from 1300 to 4350 m, reflective of the HSR thresholds used, and have a relative HSR distance of 33 ± 6 m·min^−1^ (Table 4). Players perform up to 295 HSR efforts within a match with approximately 1.6–3.2 efforts per minute [15, 38–40]. There is conflicting research regarding whether high calibre or low calibre players complete more HSR, with one study reporting similar results [39], one reporting low calibre players cover more [38], and another study reporting high calibre players cover more [40]. Studies investigating playing positions demonstrate that nomadic players cover greater absolute and relative HSR distances than key position players [5, 16, 41, 42, 44, 50]. One study investigating the influence of score margin on HSR outputs reported that during close and losing quarters HSR activity was greater than during quarters won by large margins (> 19 points) [7]. However, this was contradicted by one study which reported score margin had trivial effects on HSR outputs [44]. When accounting for possession phase, time spent at HSR (> 14 km·h^−1^) without possession was significantly greater in quarters won than quarters lost [50]. Furthermore, longer on-field stint durations and greater TD covered during a stint have been shown to negatively influence absolute and relative HSR distances [23, 44, 45]. When investigating the HSR outputs of players throughout a match, studies are agreed that HSR outputs decrease with each quarter played [44, 47, 48]. Lastly, studies examining HSR throughout a season demonstrated HSR outputs remain stable from early to late stages [42, 49], although one study reported HSR increased by ~ 10% during finals [49], while another reported a reduction in HSR distances (-9.9%) covered during the finals [44].

#### Peak Requirements

Various methods have been used to determine the peak requirements on players during AFL matches. Using a rolling window approach, peak 3-min relative distances ranged from 160 to 175 m·min^1^ for both less (5 years) experienced players [51], while 1-min peak periods are reported to be 199–223 m·min^1^ [52]. Longer periods (10 min) show most playing positions cover similar relative distances (138–141 m·min^1^), except for tall forwards who have the lowest peak requirements (131 m·min^1^) [52]. The greatest peak HSR relative distances (using a 1-min rolling window) are covered by small forwards (110 m·min^1^), closely followed by midfielders, small backs, and tall forwards (94–95 m·min^1^) [52]. During rolling durations of 1–6 min, players involved in a greater number of disposals (gaining possession and passing to a teammate) achieved lower peak running relative distances [18]. However, during longer rolling periods (7–10 min), there is an increase in relative distances covered when players have fewer disposals (1–3), before steadily lowering the more touches of the ball a player has [18]. When investigating peak relative distances covered in a match throughout an AFL season, outputs have been demonstrated to remain stable [53].

#### Technical Requirements

Studies examining the technical requirements of AFL match-play have typically used Champion Data statistics to report comparisons between calibre of players, playing positions, efficiency of various skill measures and which technical measures associate to match performance (Table 2). These studies showed that players are typically in possession of the ball for less than two seconds at a time and record on average 0.16 disposals per minute (n·min^−1^) of which kicks (0.10 n·min^−1^) are more prominent than handballs (0.06 n·min^−1)^ [6, 38–40, 54]. Studies reporting the efficiency of skills in the AFL, report that handballs are the most efficient skill, hitting the desired target 84% of the time [21], while AFL teams average a goal conversion rate of 55% [55]. One study demonstrated there is high match-to-match variability for skill involvements, with greater variability in handballs (44–63% coefficient of variation, CV) than kicks (34–52% CV) [56]. Three studies investigating comparisons between high calibre and low calibre players reported high calibre players have more disposals per minute (0.26 vs. 0.12 n·min^−1^) and cover significantly less (42–69%) distances per involvement of the ball [38–40]. Similarly, nomadic players have been reported to have more disposals per minute than key position players (0.17 vs. 0.11 n·min^−1^) [41], and when accounting for playing experience, more experienced players (> 5 years at AFL), regardless of position, have greater skill involvements during and subsequently after peak periods of play [51]. Hit-outs, clearances, and inside 50 counts were associated with ladder position in one study [57], while in their raw (absolute) form, inside 50 marks, contested possession, number of goal scorers, and higher team median disposal counts associated with desirable match outcomes [19, 22]. Alternatively, in their relative (difference to opposition) form, rebound 50 s, meters gained, kicks, and inside 50 counts associated with desirable match outcomes [9, 22, 58, 59]. The description of each technical measure is presented in Table 5.

**Table 5:** Description of technical measurements as outlined by Champion Data [69]

Technical measurement	Description
Clearance	Credited to the player who has the first disposal that clears the stoppage area
Contested possession	Possession obtained during a contest or physically pressured situation
Disposal	Summation of kicks or handballs
Disposal efficiency	Summation of kicks and handballs that hit their target
Effective handball	A handball to a teammate that hits the intended target
Effective kick	A kick of more than 40 m to a 50/50 contest or better for the team or a kick of less than 40 m that results in the intended target retaining possession
Goal conversion	Shot that resulted in a goal
Goal conversion rate	Summation of shots that resulted in a goal
Handball	Disposing of the ball with a closed fist while it rests on the opposing hand
Hit-out	Knocking the ball out of a ruck contest following a stoppage with clear control
Inside 50 m count	Number of times the ball entered the attacking 50 m zone
Kick	Disposing of the ball with any part of the leg below the knee including kicking the ball off the ground
Mark	Attaining possession by catching the ball from a kick that has travelled minimum 15 m before it touches the ground or is impeded by an opposing player
Meters gained	Net distance a team moves the ball towards their goal by either running, kicking or handballing
Player rank	Scientifically derived, objective measure of player performance weighted in favour of effective ball use and winning the disputed ball
Rebound 50	Moving the ball from the defensive 50 m zone into the midfield or attacking 50 m zone
Tackle	Using physical contact to prevent an opposition player in possession of the ball from getting an effective disposal
Time in possession	Total duration a team is in possession for the match
Turnover forced score	Scoring as a result of forcing a turnover from the opposition

#### Tactical Requirements

Three methods have been used to examine the tactical strategies of various teams in the AFL, shown in Table 2. One study utilised spatiotemporal data to examine how specific match contexts—field position and phase of play—influence team collective behaviours [30]. Three studies utilised complex network analysis (CNA) to examine the passing interactions within a team [26, 31, 60], while three studies utilised social network analysis (SNA) to identify the relationships between particular players in a team, providing insight into the functionality and efficiency of a group [2, 9, 32]. The key variables of these three analysis methods and their descriptions are shown in Table 6. Spatiotemporal data highlight that field position has more of an influence on the x-axis centroid, while phase of play has more of an influence on the width, length, and surface area covered by a team [30]. The majority of CNA studies show successful teams display more measures of clustering coefficients, centrality measures, and team entropy, where unpredictability of ball movement (e.g. who a player will pass to) and less reliance on a small number of players resulted in greater team performance [26, 31]. Studies using SNA report an association between edge count, transitivity, edge density, and match performance [9, 32], and that team selection has an impact on the final score margin [2]. This indicates that teams need their superstars (i.e. elite players), but for a greater chance at team success need a more even contribution from all players. Both network analysis methods identified that greater scoring outcomes are associated with smaller average path lengths and eigenvector centrality measures [26, 32]. One study looking at network measures initiated from kick-ins demonstrated that network characteristics do not differ between successful and unsuccessful teams; however, teams displaying lower density and higher entropy had more desirable outcomes (leading to a score) following a kick-in [60].

**Table 6:** Description of tactical analysis key variables as reported in the literature [26, 30, 32]

Tactical measurement	Description
*Collective behavioural variables*	
*x*-axis centroid	Mean longitudinal position of all players
*y*-axis centroid	Mean transverse position of all players
Length	Distance between the most forward and most backward player
Width	Distance between the two most lateral players
Surface area	Total space covered by a single team
*Passing network variables*	
Average path length	Average number of passes that occur between all possible pairs of players
Betweenness centrality	The extent to which a team’s passing network relies on particular players
Closeness centrality	How well-connected and central a player is within the teams passing structure
Clustering coefficient	The extent to which a player passes with a particular set of players
Degree centrality	The number of players that each player within the team has a direct (i.e. 1 pass) connection to
Entropy	The unpredictability of who a particular player will pass to
Edge count	Total number of interactions between players via effective passes
Edge density	Number of connections between players via effective passes, relative to the total number of possible connections
Eigenvector centrality	Dependence of a team to rely on a small group of players that have a large number of interactions with a large number of other players
Out-degree (in-degree)	Number of different players a particular player has either passed to or received a pass from
Out-strength (in-strength)	Number of passes (made or received) made by a player
Transitivity	The number of triads in a team, in proportion to the total possible number of triads. A triad represents the concept that two players are connected via a third player

#### Interaction of Match-Play Elements

Studies that analysed physical and technical elements of match-play typically examined the association between physical measures and skill involvements [16, 23, 41], their relationship to player performance measures [4, 16, 17, 23, 38], and how score margin influences both elements [7]. Likewise, studies investigating technical and tactical elements have demonstrated how technical skill measures are mediated by tactical strategies [8] and the contribution each element has to match outcome [9]. These studies typically isolate and compare elements of match-play as opposed to integrating and understanding their relationship to one another. Recent research has looked to integrate data sources by examining physical and technical [61], physical and tactical [25], and a combination of all three elements [62] during individual possession chains. Combined, these studies demonstrated that when accounting for technical skill involvements (kicking, handballing, and pressure applied) [61, 62], and starting field location [25], attacking (with the ball), and defensive (without the ball) possession chains have similar activity requirements. Additionally, compared to stoppages, possession chains initiated from a turnover or kick-in involved the most TD and HSR [25, 62].

## Discussion

This systematic review summarised three elements of AFL match-play and outlined how recent research has looked to integrate data from multiple elements. Forty-eight studies were identified to have analysed the physical, technical, and/or tactical elements of AFL match-play. While physical and technical elements have been studied extensively, tactical elements have only recently been investigated with eight of the nine studies identified in this review conducted since 2018. Furthermore, elements of match-play are typically analysed in isolation, with few studies (*n* = 16) incorporating more than one construct. To date, only two studies have investigated the influence that tactical elements have on the activity requirements of AFL athletes, and only one study has integrated data sources from all three elements of match-play.

### Summary of Physical Elements

The present systematic review showed that physical requirements of match-play are the most commonly investigated element of AFL performance. While there are out-of-game contextual factors (e.g. travel and sleep quality) that can affect absolute and relative distances covered in a match [63], they were outside the scope of this review. Playing position and the calibre of player were the main comparisons (*n* = 10) undertaken by studies within this review. Seven studies, which all examined different teams within the AFL, identified nomadic players as covering the greatest absolute and relative distances [5, 15, 23, 41–44]. Despite nomadic players spending less time on the ground [5], given their tactical roles within the team (the link between the offence and defence) allowing them to cover greater distances, and the observation that they are the most rotated group (allowing greater recovery from transient fatigue) [23, 42] the findings are unsurprising. Similarly, three studies reported high calibre players cover greater TD, although both high calibre and low calibre players cover similar relative distances [38–40]. High calibre players are generally older, more experienced and are on the ground for longer periods of time (106 min vs. 96 min) [38, 51]. As such, high calibre players cover greater absolute distances, but run at similar relative distances to lower calibre players [38, 40]. Absolute and relative distances covered in a match may be linked to the team’s ranking in the competition [47, 48]. One study investigating a lower rank team (bottom 25%) reported a reduction in TD covered in the last quarter compared to the first [48], whereas one study investigating a higher ranked team (top 25%) reported no significant differences between the TD covered from the first to last quarter [47]. Individual studies have shown that higher rank teams in both AF and soccer have been shown to be more economical with their running due to superior fitness, more technical proficiency, and greater tactical knowledge [47, 64, 65]. Predominantly, relative distances covered during a match have been linked with match significance [42, 49], detailing that relative distances are highest at the terminal end of the season, and increase a further 11% during the finals campaign [49]. As the finals are made up of the best eight performing teams of the year, relative distances may be increased due to the quality of opposition, with one study in AF indicating an association between finals matches and physical activity requirements [42]. However, recently, research has reported a *decrease* in relative distances covered during the finals [44], potentially being explained by the recent shift in AFL tactics, evolving from a possession style (passing to an unobstructed teammate) to a repossession style (characterised by more contested and congested play) [8], which results in less TD being covered [42].

AFL players typically cover most distance at low speeds (< 14 km·h^−1^); however, this is punctuated by intermittent bursts of HSR (> 14 km·h^−1^) (Table 4). Similar to absolute and relative TD covered, nomadic and high calibre players were shown to spend more of the match at high speeds than their key position and low calibre counterparts [5, 16, 38–40, 44, 50]. However, this is in contrast with other studies that demonstrated low calibre players spend more or at least similar match times at high speeds [38, 39]. These inconsistencies in results demonstrate that HSR requirements may be reflective of tactics employed [66], the demographic of the playing list (e.g. proportion of high-to-low calibre players) [38], opposition strength [42], and the measurement error of different GPS units [13]. This highlights that HSR covered during a match is dependent on numerous variables and although player comparisons during match-play analysis may provide useful information, they have limitations and therefore should be interpreted cautiously when considering these contextual factors. Furthermore, irrespective of score margin, when the team is not in possession of the ball HSR is greater, suggesting that defensive phases of play are more physically demanding than attacking phases [50]. Despite team rankings having a potential effect on the TD covered during each quarter, when accounting for HSR, all teams investigated showed a decline in their HSR outputs after each quarter played [44, 47, 48]. However, from a relative standpoint, higher (also fitter) ranking teams may be better equipped to recover at a faster rate from the transient fatigue associated with HSR, though this requires further investigation. Lastly, although HSR remains stable across the season [42, 49], conflicted information exists regarding HSR during the finals, with one study reporting an increase in HSR [49], and one study reporting a decrease in HSR [44]. Studies in rugby league have reported matches against stronger opposition have small-to-moderate increases in HSR distances [67]. However, other studies in rugby have demonstrated that defensive play has a greater impact on world cup finals compared to attacking play [68]. This may explain the decrease in HSR activity during finals as increases in defensive play could lead to more stoppages and congestion which has been previously shown in AF to reduce player activity [42].

Peak periods of play have been associated with the most crucial moments in a match and therefore have recently been investigated in AF to add more specificity to training design and prescription [18, 51–53]. The use of ‘peak periods’ analysis has been undertaken using various methods. Using fixed 3-min windows, there is no influence of player experience for peak speeds during a match [51]. However, experienced players demonstrated greater running outputs following peak (sustained high intensity) passages of play [51], suggesting that experienced players are more equipped to tolerate the transient fatigue associated with peak periods of play due to their longevity in the AFL system. Similar to global physical demands of match-play, the tactical role of nomadic players requires them cover greater peak relative distances and peak HSR distances [52]. Only two of the studies combined peak period activity requirements with the technical involvements of players [18, 53] finding players typically have lower physical output the more skill involvements they have. However, studies examining peak requirements only account for a small portion of match time, neglecting other important phases of a match. Therefore, combining technical and physical data sources during greater time periods (e.g. quarter or possession chains) warrants further investigation. This information may assist practitioners in understanding how skill involvements influence activity requirements and inform representative drill design. However, many of studies in this review used data from a single team and season of AFL; in terms of representativeness, the information would be more insightful if the data covered a greater number of years and teams. This would help reduce the risk of reporting any anomalies that may have potentially been specific to that year and/or players at the time of the investigation.

### Summary of Technical Elements

Technical output was identified as being influenced by the calibre of player [38–40] and the individual’s playing position [41] in this review. Nomadic players have greater disposals per minute than key position players [41]. This was explained as being related to the tactical role of nomadic plays (i.e. they are linking players to the offence and defence and are the distributors of the ball following a stoppage in play), which allows these individuals to gain more possession than key position players. Alternatively, high calibre players travel less distance per disposal [38–40], suggesting that better performing athletes have greater match awareness and are able to have lower overall physical output while having a positive influence on the match. While research demonstrated that higher calibre and nomadic players have greater activity requirements [5, 15, 38–43], no research has examined the influence that specific technical skill involvements (i.e. kicks or handballs) have on the physical output of players in AF. One study reported the distribution of physical and technical output in various possession chains [61]; however, the specific influence of skill-based match events on the physical output of players during competitive matches remains unclear. This type of research is important in understanding the relationships between various possession types and the activity requirements of AFL athletes. A lack of context in athlete activity outputs may limit a practitioner’s ability to design training drills, tactical strategies and analyse player performance. For example, with knowledge of how an athlete’s physical output is impacted by applying both physical (tackling) and perceived pressure (closing down on an opponent) on the opposition, coaches can begin to replicate those requirements in training to ensure their players are adapted to sustain desired levels throughout a match.

The present review showed numerous skill measures demonstrate associations with match performance. Indeed, it has been suggested that coaches should focus on winning clearances (starting play from an attacking field position) and setting up attacking structures that generate more repeat entries and marks inside 50 [9, 22, 57–59]. Additionally, teams should aim to decrease predictability of ball movement by spreading the ball amongst players and having multiple targets for goal rather than one or two specific players, making it harder for the opposition to defend [19]. Collectively, these studies highlight the need for combining technical and tactical analysis so that coaches can understand the efficiency of their team’s ball movement and if the team’s technical output is reflective of the way in which the team wants to move the ball. Additionally, with the integration of technical and tactical data, coaches can analyse opposition tactics and begin to design defensive structures that will prevent scoring opportunities for the opposition, while simultaneously setting up tactics that can exploit the opposition during attacking phases.

### Summary of Tactical Elements

The three identified tactical analysis methods reported in this review allow coaches to analyse and subsequently devise and implement tactical strategies aimed at enhancing performance based on the information presented. Using the results of the study examining spatiotemporal data [30], understanding that player density increases when the ball is located within scoring positions on the field, coaches can implement tactics that aim to increase the attacking team’s surface area in order to spread the defence and create more space closer to goal. Likewise, using the result of the network analysis studies [2, 9, 26, 31, 32, 60] coaches can implement tactics characterised by unpredictable and faster ball movement to try to increase their chances of winning matches [9, 26, 31, 32]. However, while this review demonstrates that the use of the identified tactical analysis methods can be employed to examine the collective behaviours and passing networks of AFL teams, it is understood that in isolation these insights are limited. Therefore, to enhance our understanding of tactics resulting in desirable outcomes, future research should combine tactical analysis methods (network analysis, spatiotemporal analysis) with physical and/or technical data. This could involve examining how different game styles, which emphasise slower ball movement (e.g. kick and mark) as opposed to fast ball movement (e.g. kick and handball), disrupt opposition defensive structures and create more scoring opportunities. Coaches could then combine these findings with the physical requirements associated with each game style to ensure their players are physically equipped to implement these tactics.

### Influence of Integrated Data Sources

The majority of studies identified in this review that have integrated data sources from multiple elements examined associations between specific physical and technical indicators and individual and team performances [4, 16, 17, 23, 38]. While these studies have highlighted the importance of technical aspects and match performance, having a focus on generating marks inside the forward 50 (50 m space within proximity of the goals), clearances, and less reliance on particular players, they have not examined the interaction between both the physical and technical elements on match-play performance. Physical, technical, and tactical elements are inextricably linked; however, the relationship between these may be affected by different contextual factors (e.g. tactics implemented, the phase of play, or field location) within a match and therefore should be investigated accordingly. Recent research examining individual possession chains has reported associations between these different elements by integrating data from multiple sources [25, 61, 62]. While previous research has linked greater skill involvements with lower physical output [18], when separated into attacking and defensive possession chains, similar physical outputs are observed [25, 61, 62], despite the greater skill involvements during attacking chains [61]. Additionally, events prior to possession demonstrated to influence activity requirements, where plays initiating furthest from a team’s own goal and following an intercept (a turnover of possession) increased players TD and HSR outputs [25]. Collectively, these studies signify the need for more possession chain analysis which easily allows for integration of all three elements and is more useful to coaches when designing tactics and analysing player match performance compared to whole, half or quarter match activity analysis. Coaches can use possession chain analysis to design tactics (e.g. quick transitioning from defence to attack) based on the activity requirements associated with different technical skill measures (e.g. kicks vs. handballs or player involvements) and additionally analyse player behaviours during various tactical scenarios (e.g. following a turnover). This, in comparison with previous isolated studies, would provide greater insights into match performance, as well as provide practitioners with a more comprehensive data source for training drill design and prescription.

### Limitations

A limitation of this review was the lack of homogeneity in the HSR thresholds amongst studies. Among the 30 studies investigating physical requirements, six different thresholds were used for the term HSR ranging from 14 to 20 km·h^−1^. Additionally, different models of GPS units were utilised, posing further issues with comparative analysis due to the dissimilarity in the hardware and satellite systems used (e.g. sampling frequency, GPS vs. global navigation satellite system), the greater error in the earlier hardware, and the software (i.e. algorithms to smooth data) used to collect and analyse the data. Furthermore, only two studies in this review used multi-team analysis for physical requirements of match-play [43, 44], limiting the understanding of how match activities are influenced by unique tactical strategies of various teams and the characteristics (e.g. level of experience, age, fitness levels) of a team. Additionally, despite recent research integrating data from multiple elements, only one study incorporated all three elements of match-play.

Another limitation of this review includes the analysis methods undertaken by most studies. Many studies in this review used analysis methods (e.g. repeated measures) that are more susceptible to bias, do not account for levels of clusters (e.g. hierarchical data), and are unable to handle common analytical issues such as missing data, potentially leading to misinterpretation of the results of these studies. Furthermore, although difficult in applied field studies, lack of consistency of methods, positional groups, speed thresholds, and skill variables measured prevented a meta-analysis from being conducted. Consistency across studies would allow direct comparisons between studies and develop normative values. Lastly, most studies in this review failed to account for in-game contextual factors that can influence the activity requirements of players during match-play such as field location or the phase of play. This provides practitioners with no context into how activities occur, making difficult to analyse match performance and design training drills representative of match context.

### Future Directions

Future research should aim to analyse all three elements of match-play in cohesion to develop a holistic understanding of match activity requirements. To account for analytical limitations in previous studies, it is essential that appropriate methodologies such as mixed models be used in future physical activity analyses [23, 25]. Future research should continue to expand on possession chain analysis, which provides a more in-depth understanding of the activity requirements occurring in match-play compared to whole, half, or quarter match analyses. This research should be expanded by analysing and integrating other possession chain factors such as the skill-based match events (e.g. kicks, handballs, marks, pressure) occurring in various possession chains. This would assist coaches in player analysis, as well as in designing training drills that are representative of specific match context.

## Conclusion

Understanding how the physical, technical, and tactical aspects of AF match-play are related is important for designing match strategy and training design. This review has described the global and peak running requirements of AFL match play, the frequency and efficiency of various technical skills, contextual factors that influence both technical and physical activities, and the common tactical analysis methods utilised in the AFL. Match running requirements in the AFL are reliant on numerous technical and tactical variables that are uniquely different depending on the team investigated, context of match-play, and the competition being played against. However, this review highlighted that despite an extensive body of literature describing match locomotive activities, there is a lack of data surrounding the influence that technical and tactical variables have on the physical output of AFL athletes. Future investigation into this will give greater insight into the physical requirements associated with various tactical strategies that emphasise particular technical skills, which, in turn, will provide a deeper understanding into match performance and provide practitioners with more comprehensive data for player analysis and for guiding training design in the AFL.

## Data Availability

Not applicable.

## Abbreviations

AF: Australian Football
AFL: Australian Football League
AFLW: Australian Football League Women’s
CL: Confidence limits
CNA: Complex network analysis
CV: Coefficient of variation
GPS: Global positioning system
HSR: High-speed running
Hz: Hertz
PRISMA: Preferred Reporting Items for Systematic Review and Meta-analysis
SD: Standard deviation
SNA: Social network analysis
TD: Total distance
